# Characterization and phylogenetic analysis of the mitochondrial genome of *Sarcocheilichthys sinensis* (Bleeker) from Baima Hu Lake

**DOI:** 10.1080/23802359.2019.1710280

**Published:** 2020-01-14

**Authors:** Lurong Gong, Yuexi Lu, Lizhen Gu, Lihui Dong, Jiangfeng Ren, Shoubao Yang

**Affiliations:** a College of Life Sciences, Shaoxing University, Shaoxing, Zhejiang, P. R. China;; b Department of Biology, Chunhui Senior High School, Shangyu, Zhejiang, P. R. China

**Keywords:** Mitogenome, *Sarcocheilichthys sinensis*, characterization, phylogenetic analysis

## Abstract

*Sarcocheilichthys sinensis* (Bleeker), is a small-sized benthopelagic fish with ornamental value. In the present study, the complete mitochondrial genome of *S. sinensis* was sequenced and determined. The complete mitogenome of *S. sinensis* was 16,683 bp in length, consisting of 22 tRNA genes, 13 protein-coding genes, 2 rRNA genes, and 2 non-coding regions. The overall base composition of the *S. sinensis* mitogenome is 30.50% A, 26.28% T, 26.60% C, and 16.61% G, exhibiting obvious AT bias (56.79%). The phylogenetic analysis showed that *S. sinensis* clustered in genus *Sarcocheilichthys*. Present study provides useful data to population genetics and conservation biology of *Sarcocheilichthys* fishes.

The Chinese lake gudgeon, *Sarcocheilichthys sinensis* (Bleeker), is a small-sized benthopelagic fish distributed widely in various region of China (Song and Ma 1994; Zhang et al. 2008; Li et al. 2011; Zhu et al. 2017). It is an omnivorous and edible fish with ornamental value (Guo et al. 1995; Song and Ma 1996; Huan et al. 2010; Li et al. 2011). Baima Hu Lake is one of the most famous lakes in Shaoxing City, East China. Although *S. sinensis* is a common fish in East Asia (Hosoya^1982^; Barabanshchikov 2004; Yuan et al. 2010; Shen et al. 2017), little is known about it in Baima Hu Lake.

The mitochondrial genome (mitogenome) could be useful data in population genetics and conservation biology studies, due to rich signals from its gene arrangement and sequence information (Liu and Cui 2009; Chen et al. 2013; Yang et al. 2015; Cui et al. 2019).

Herein, a new complete mitogenome of *S. sinensis* (GenBank accession no. MN711646) was characterized. *Sarcocheilichthys sinensis* was collected from Baima Hu Lake, Zhejiang Province of China (33°13′47.7′′N, 119°08′49.4′′E), and preserved in 99% ethanol in Shaoxing Aquatic Service Platform (accession no. SXAF191122). Total DNA was extracted and used as template, the PCR amplification was carried out and the protocols were: initial denaturation at 94 °C for 5 min, followed by 37 cycles of amplification (93 °C for 50 s, 55–57 °C for 25 s, and 72 °C for 90 s) and 1 final cycle of 8 min at 72 °C.

The complete mitochondrial genome of *S. sinensis* is 16,683 bp in length, consists of 13 protein-coding genes (PCDs), 22 tRNA genes, 2 rRNA genes, and 2 non-coding regions. Among these genes, 12 PCDs (*ND1–5 and ND4L*, *COXI–III*, *ATP6*, *ATP8*, and *CytB*), 14 tRNA genes (*tRNA^Arg^*, *tRNA^Asp^*, *tRNA^Gly^*, *tRNA^His^*, *tRNA^Phe^*, *tRNA^Ile^*, *tRNA^Lys^*, two *tRNA^Leu(UUR)^* genes, *tRNA^Met^*, *tRNA^Ser(AGY)^*, *tRNA^Thr^*, *tRNA^Trp^*, and *tRNA^Val^*), and 2 rRNA genes (*12S rRNA* and *16S rRNA*) were encoded on the H-strand, while other genes including one PCD (ND6) were encoded on the L-strand. The gene order within the complete mitogenome of *S. sinensis* is similar to other vertebrate species (Zhu et al. 2019).

The overall base composition of the *S. sinensis* mitogenome is 30.50% A, 26.28% T, 26.60% C, and 16.61% G, respectively, exhibiting obvious AT bias (56.79%), which is similar to other vertebrate mitogenomes (Yang et al. 2015; Li et al. 2016). The *12S rRNA* gene (960 bp) and *16S rRNA* (1685 bp) gene are located between two tRNA genes (*tRNA^Phe^* and *tRNA^Leu(UUR)^*), and are separated by the *tRNA^Val^* gene, which is similar to most of vertebrates mitogenomes (Wang et al. 2016).

The phylogenetic tree was constructed using the neighbor-joining method. The results showed that *S. sinensis* is clustered with other *S. sinensis*, and clustered in genus *Sarcocheilichthys* with other *Sarcocheilichthys* fishes including *Sarcocheilichthys davidi*, *Sarcocheilichthys kiangsiensis*, *Sarcocheilichthys lacustris*, *Sarcocheilichthys nigripinnis*, *Sarcocheilichthys parvus*, and *Sarcocheilichthys variegatus* (Figure 1). While it showed distant kinship with other Cyprinidae fishes. This study provides useful data to population genetics and conservation biology of *Sarcocheilichthys* fishes.

**Figure 1.**
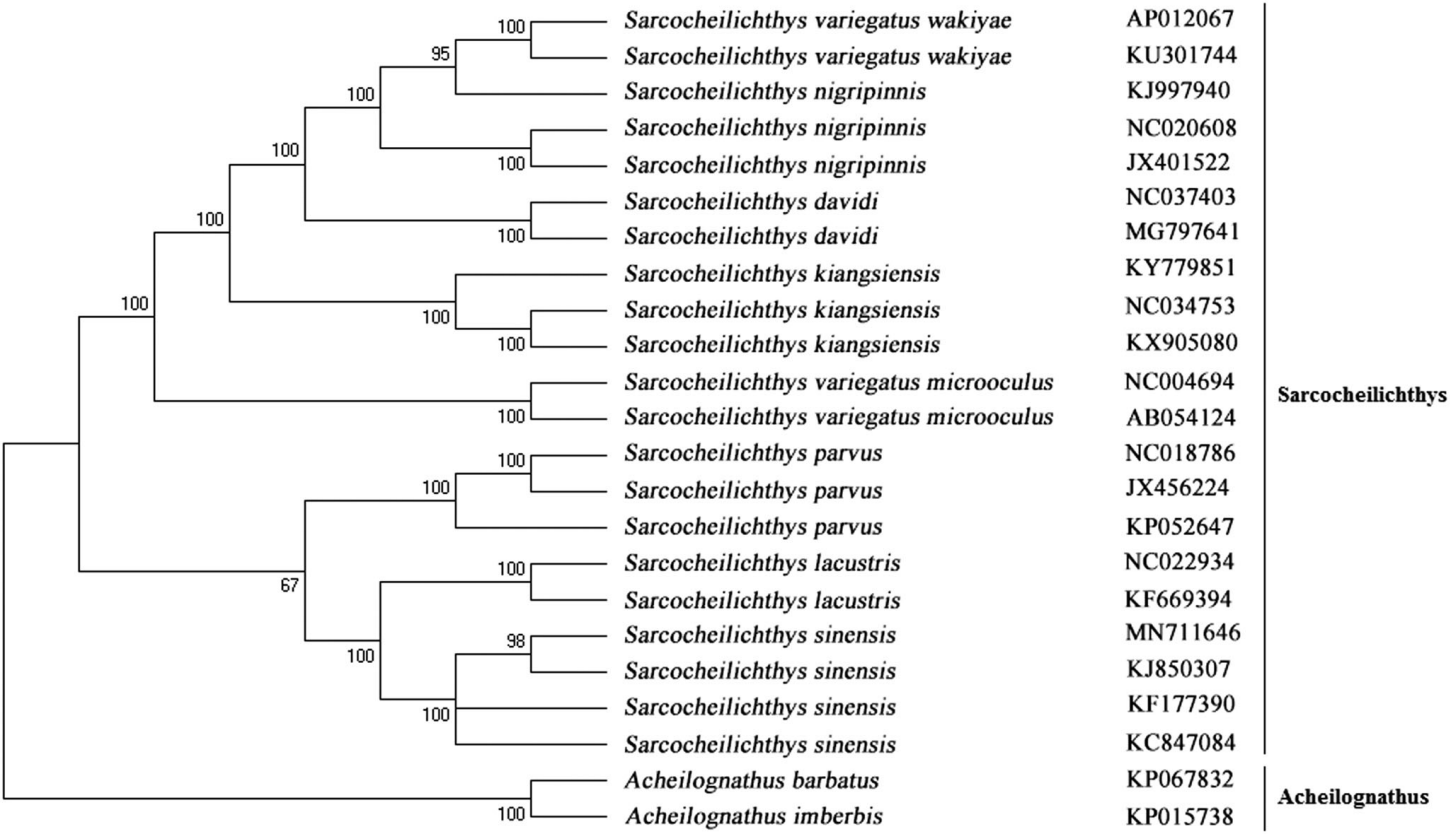
The phylogenetic analysis of *Sarcocheilichthys sinensis* and other Cyprinidae fishes based on the mitogenome sequences.
